# Effect of inhaled corticosteroids on blood eosinophil count in steroid-naïve patients with COPD

**DOI:** 10.1136/bmjresp-2016-000151

**Published:** 2016-09-08

**Authors:** James L Kreindler, Michael L Watkins, Sally Lettis, Ruth Tal-Singer, Nicholas Locantore

**Affiliations:** 1 GSK R&D, King of Prussia, Pennsylvania, USA; 2 GSK R&D, Research Triangle Park, North Carolina, USA; 3 GSK R&D, Uxbridge, UK

**Keywords:** Eosinophil Biology, COPD Pharmacology

## Abstract

**Introduction:**

Sputum and blood eosinophil counts have attracted attention as potential biomarkers in chronic obstructive pulmonary disease (COPD). One question regarding the use of blood eosinophils as a biomarker in COPD is whether their levels are affected by the use of inhaled corticosteroids (ICS), which are commonly prescribed for COPD.

**Methods:**

We performed a retrospective analysis of peripheral blood leucocytes from a previously completed clinical trial that examined effects of ICS in steroid-naïve patients with COPD.

**Results and conclusion:**

The data show that the ICS-containing treatment arms (containing fluticasone propionate) had a small effect on peripheral blood eosinophils in steroid-naïve patients with COPD.

**Trial registration number:**

NCT00358358; Post-results.

**Key messages**

- Inhaled corticosteroids have a small effect on peripheral blood eosinophil counts.
- The effect is likely small enough on average not to affect patient stratification using cut-off values.

## Introduction

Chronic obstructive pulmonary disease (COPD) is a heterogeneous disease for which there are limited choices with respect to therapeutic mechanisms of action. In order to direct patient-specific therapy in a disease with high prevalence and high clinical variability, the medical community needs to identify and scientifically validate COPD biomarkers.

Sputum and blood eosinophil counts have attracted attention as potential biomarkers. For example, higher sputum eosinophil counts in patients with COPD predicts clinical response to inhaled corticosteroids (ICS),^1^ as it does for anti-interleukin 5 (IL5) therapy in asthma.^2^ Peripheral blood eosinophil count was proposed more recently as a biomarker to direct corticosteroid therapy during COPD exacerbations^3^ and to select those who will benefit from medicines containing ICS.^4^ ^5^ Currently, blood eosinophil levels are being studied in clinical trials assessing the efficacy of anti-IL5 therapies such as mepolizumab (NCT02105948) and benralizumab (NCT02138916) in patients with COPD.

One question regarding the use of blood eosinophils as a biomarker in COPD is whether their levels are affected by the use of ICS, which are commonly prescribed for COPD. Our post hoc analysis of an interventional study in steroid-naïve patients with COPD evaluated the extent to which ICS affected blood eosinophil counts and compared the distribution of baseline eosinophil counts in the study with the COPD and non-COPD control cohorts from (Evaluation of COPD Longitudinally to Identify Predictive Surrogate End points) ECLIPSE.

## Methods

### Study design

SCO104925 (NCT00358358) was a 12-week, double-blind, parallel-group study comparing patients randomised to receive placebo, fluticasone propionate [FP]/salmeterol combination (FSC) or the individual components of FSC (FP or salmeterol). Individuals were included if they had COPD as defined by a post salbutamol forced expiratory volume in 1 s (FEV_1_)/forced vital capacity (FVC) ≤70% and a post salbutamol FEV_1_ ≥30% and ≤70% of predicted. Individuals were excluded if they used oral corticosteroids or ICS-containing products for more than 14 consecutive days in the prior 6 months or at any time within 30 days of screening.

In this post hoc analysis, we examined blood eosinophil counts from complete blood counts that were measured at baseline, and week 6 and 12, providing an opportunity to examine the impact of steroid-containing therapies on blood eosinophils in a steroid-naïve COPD population.

The patients for this post hoc analysis consisted of those persons who completed the study and had eosinophil levels available for all time points. Additionally, to eliminate potential confounding, persons who had an exacerbation or received oral corticosteroids during the study period were excluded.

The design of the ECLIPSE observational study (SCO104960, NCT00292552) has been published elsewhere.^6^ Briefly, patients with COPD had a smoking history ≥10 pack-years, a post-bronchodilator FEV_1_/FVC ratio <0.7 and an FEV_1_ <80% of predicted. Controls (with or without smoking history) had FEV_1_/FVC >70% and FEV_1_ >85%.

Both studies were sponsored by GSK and conducted according to Good Clinical Practice and in accordance with guidelines from the Declaration of Helsinki. Each study protocol was approved by relevant ethical boards and institutional review boards at participating centres.

### Statistical methods

Owing to the small sample size for the study and the presence of outliers, summaries for continuous variables are provided as median and IQR; categorical summaries are presented as percentages. Hodges-Lehmann estimates^7^ and associated 95% CIs for the difference in location shift of changes in blood eosinophil counts between the ICS-treated and non-ICS-treated groups were calculated based on Wilcoxon rank-sum tests. The comparisons of the baseline distribution of blood eosinophil counts for SCO104925 and the ECLIPSE cohorts used two-sample Kolmogorov-Smirnov tests.

## Results

Overall, 161 persons were randomised to treatment in study SCO104925, with 80 assigned to non-ICS-containing treatments (salmeterol or placebo) and 81 to ICS-containing treatments (FSC or FP). One hundred and fourty-three patients completed the 12-week study; 73 from the non-ICS-treated patients and 70 from the ICS-treated patients. To remove potential confounding factors, patients who either had an exacerbation and/or were prescribed oral corticosteroids during the treatment period were excluded. Accounting for these factors, 59 patients from the non-ICS-treated arms and 53 patients from the ICS-treated arms (112 total patients) were used in this post hoc analysis.

The primary and key secondary outcome measures for SCO104925 measured the impact of ICS on impulse oscillometry and lung imaging end points in steroid-naïve patients with COPD.^8^ These results are not germane to this retrospective analysis of blood eosinophils and, therefore, will not be further discussed.

### Changes in blood eosinophil counts

In SCO104925, median (IQR) blood eosinophil count was 200 (120 to 310) cells/μL at baseline for patients who received ICS during treatment, and 230 (120 to 410) cells/μL for patients not receiving ICS during treatment. Median (IQR) changes in eosinophil count for ICS-treated patients were −30 (−90 to 10) cells/μL at week 6 and −30 (−90 to 20) cells/μL at week 12, while median (IQR) changes for non-ICS-treated patients were −20 (−80 to 70) cells/μL at week 6 and 10 (−80 to 80) cells/μL at week 12. The differences in eosinophil count changes between groups were not significant at week 6 (median (95% CI) difference −20 (–60 to 20); p=0.315) nor at week 12 (median (95% CI) difference −40 (80 to 10); p=0.090). However, this may be due to lack of power for these comparisons based on the size of the study. Study characteristics and eosinophil levels are shown in table 1.

**Table 1:** Baseline patient characteristics and eosinophil counts during the study

Baseline characteristics	Non-ICS arms (n=59)	ICS arms (n=53)
Age, years	66 (61 to 71)	64 (54 to 71)
Male, %	80	74
Smoking history, pack-years	46 (33 to 55)	42 (26 to 60)
Current smoker, %	51	55
Post-bronchodilator FEV_1_, L	1.40 (1.07 to 1.65)	1.50 (1.15 to 1.87)
Post-bronchodilator FEV_1_ per cent predicted	50 (40 to 61)	55 (45 to 63)
Eosinophil counts, cells/μL	Non-ICS arms (n=59)	ICS arms (n=53)
Eosinophil count at baseline	230 (120 to 410)	200 (120 to 310)
Change, week 6	−20 (−80 to 70)	−30 (−90 to 10)
Change, week 12	10 (−80 to 80)	−30 (−90 to 20)
Percentage of patients with eosinophil count at baseline ≥150	71	66
Percentage of patients with eosinophil count at baseline ≥300	34	26

Values presented as median (IQR) unless otherwise noted.

FEV1, forced expiratory volume in 1s; ICS, inhaled corticosteroids; IQR, interquartile range.

### Comparison of baseline distribution of blood eosinophil counts

**Figure 1:**
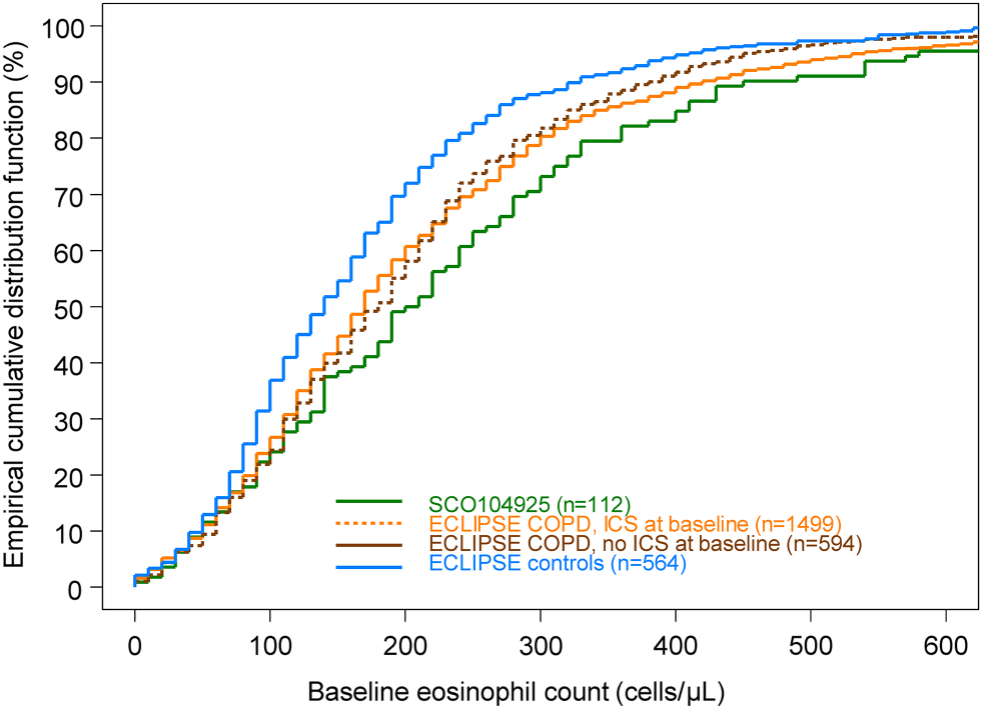
Empirical distribution function for SCO104925 patients and the ECLIPSE cohorts.

To contextualise the findings from SCO104925, the distribution of baseline blood eosinophil counts in SCO104925 patients was compared with those in the ECLIPSE study. The baseline eosinophil counts of non-COPD controls in the ECLIPSE study were distributed differently (lower values) than the eosinophil counts of patients with COPD in ECLIPSE and SCO104925 (p<0.001 for both comparisons with controls). The distribution of blood eosinophils for SCO104925 patients was not significantly different from either the ECLIPSE COPD cohort reporting ICS use (p=0.102) or those not reporting ICS use (p=0.137). Consistent with ICS having a small effect on blood eosinophil counts, there was no significant difference in the distribution of blood eosinophil counts in ECLIPSE patients reporting ICS use at baseline compared with those patients not reporting ICS use (p=0.246; figure 1). Summary statistics for each cohort are shown in table 2.

**Table 2:** Summary of eosinophil counts at baseline for the ECLIPSE study

Baseline eosinophils, cells/μL	COPD: ICS use at baseline (n=1499)	COPD: no ICS use at baseline (n=594)	Non-COPD controls (n=564)
Eosinophil count, median (IQR)	180 (110 to 290)	190 (120 to 270)	150 (90 to 230)
Eosinophil count ≥150 cells/μL, n (%)	918 (61)	374 (63)	290 (51)
Eosinophil count ≥300 cells/μL, n (%)	347 (23)	121 (20)	73 (13)

COPD, chronic obstructive pulmonary disease; ECLIPSE, Evaluation of COPD Longitudinally to Identify Predictive Surrogate End points; ICS, inhaled corticosteroids; IQR, interquartile range.

**Figure 2:**
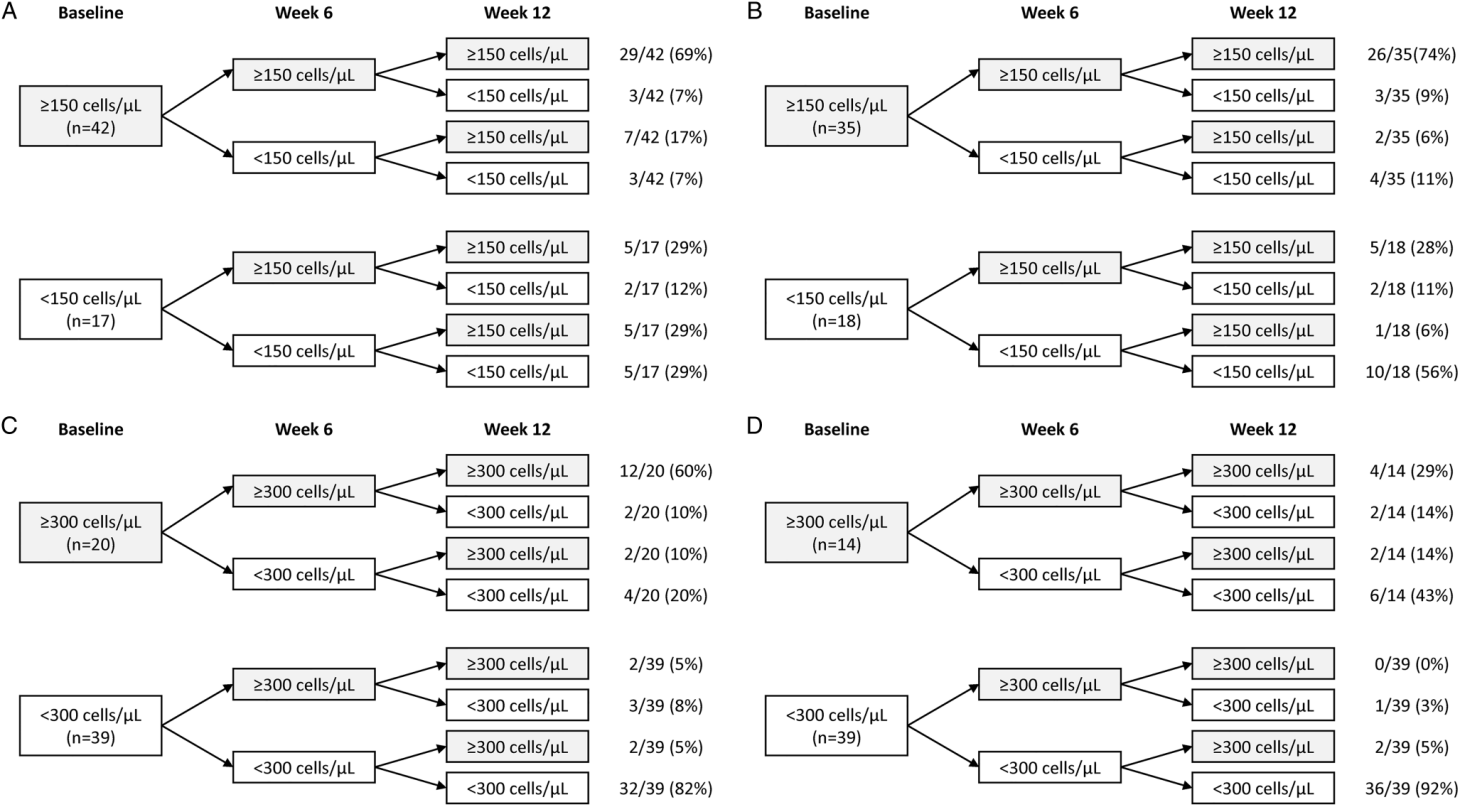
Peripheral blood eosinophil levels based on a threshold of (A) 150 cells/μL, patients not randomised to inhaled corticosteroids (ICS)-containing treatment (n=59), (B) 150 cells/μL, patients randomised to ICS-containing treatment (n=53), (C) 300 cells/μL, patients not randomised to ICS-containing treatment (n=59) and (D) 300 cells/μL, patients randomised to ICS-containing treatment (n=53).

As some of the recent discussion regarding blood eosinophils as a biomarker to direct treatment revolves around threshold values, we investigated changes in peripheral blood eosinophil levels based on thresholds and found that although the majority of patients remain in their baseline category, there is some regression to the mean with respect to using thresholds (figure 2).

## Discussion

The desire to practice precision medicine has prompted a broad, intensive search for biomarkers that can be used to direct personalised therapy in COPD. Eosinophils have been part of this search for more than a decade.^9^ Much of the early focus was on sputum eosinophils as a direct marker of lung inflammation. The data presented here do not directly address the effect of ICS on sputum eosinophils, as sputum was not collected in the SCO104925 trial. However, there are data to suggest that changes in sputum eosinophil count due to titration of ICS therapy^10^ or discontinuation of ICS therapy^11^ correlate with COPD exacerbations, although not all studies have shown a significant change in sputum eosinophils in response to ICS therapy in patients with COPD.^12^ As measuring sputum eosinophils can be technically difficult and may not be feasible for many practitioners caring for patients with COPD, there is interest in blood eosinophils as a more practicable alternative. The potential importance of blood eosinophils as a biomarker is highlighted by recent clinical trial data.^4^ ^5^ The data presented here show ICS-containing treatments having a small effect on blood eosinophil levels in steroid-naïve patients. The lack of a pronounced effect suggests that peripheral blood eosinophil level reduction per se may not be the mechanism for the clinical efficacy seen with ICS in COPD, despite peripheral blood eosinophils being identified as a potential biomarker for enhanced exacerbation reduction with inhaled and oral corticosteroid-containing medications.
